# Amphibian and reptile road-kills on tertiary roads in relation to landscape structure: using a citizen science approach with open-access land cover data

**DOI:** 10.1186/s12898-017-0134-z

**Published:** 2017-06-26

**Authors:** Florian Heigl, Kathrin Horvath, Gregor Laaha, Johann G. Zaller

**Affiliations:** 1 Institute of Zoology, University of Natural Resources and Life Sciences, Vienna, Gregor Mendel Straße 33, 1180 Vienna, Austria; 2 Institute of Applied Statistics and Computing, University of Natural Resources and Life Sciences, Vienna, Peter Jordan Str. 82, 1190 Vienna, Austria

**Keywords:** Anurans, Kernel density estimation, Landscape ecology, Participatory science, qgis, Road mortality, Snakes

## Abstract

**Background:**

Amphibians and reptiles are among the most endangered vertebrate species worldwide. However, little is known how they are affected by road-kills on tertiary roads and whether the surrounding landscape structure can explain road-kill patterns. The aim of our study was to examine the applicability of open-access remote sensing data for a large-scale citizen science approach to describe spatial patterns of road-killed amphibians and reptiles on tertiary roads. Using a citizen science app we monitored road-kills of amphibians and reptiles along 97.5 km of tertiary roads covering agricultural, municipal and interurban roads as well as cycling paths in eastern Austria over two seasons. Surrounding landscape was assessed using open access land cover classes for the region (Coordination of Information on the Environment, CORINE). Hotspot analysis was performed using kernel density estimation (KDE+). Relations between land cover classes and amphibian and reptile road-kills were analysed with conditional probabilities and general linear models (GLM). We also estimated the potential cost-efficiency of a large scale citizen science monitoring project.

**Results:**

We recorded 180 amphibian and 72 reptile road-kills comprising eight species mainly occurring on agricultural roads. KDE+ analyses revealed a significant clustering of road-killed amphibians and reptiles, which is an important information for authorities aiming to mitigate road-kills. Overall, hotspots of amphibian and reptile road-kills were next to the land cover classes arable land, suburban areas and vineyards. Conditional probabilities and GLMs identified road-kills especially next to preferred habitats of green toad, common toad and grass snake, the most often found road-killed species. A citizen science approach appeared to be more cost-efficient than monitoring by professional researchers only when more than 400 km of road are monitored.

**Conclusions:**

Our findings showed that freely available remote sensing data in combination with a citizen science approach would be a cost-efficient method aiming to identify and monitor road-kill hotspots of amphibians and reptiles on a larger scale.

## Background

Amphibian and reptile species are endangered worldwide, suffering from numerous threats such as habitat modification and fragmentation, diseases, pollution, invasive species or climate change [1–4]. In Austria, where the current study was conducted, all 20 amphibian species and all 14 reptile species are protected by national conservation laws [5]. Focusing on habitat fragmentation, roads can have various negative effects on many vertebrate species [6–9]. The most direct negative effect of road traffic on animal populations is through fatal collisions with vehicles, i.e. road-kill [2]. Road-kill does not affect all taxonomic groups in the same way. Amphibians (toads, newts and salamanders) are mostly affected by road-kill when crossing roads during migration between their breeding and hibernation sites; reptiles are even attracted by the favourable microclimate on roads [4, 8, 10–12]. Amphibians and some reptile species are even more susceptible to road-kill because they get immobile in response to an approaching vehicle [13, 14]. As mentioned by Rytwinski and Fahrig [15] there are relatively few studies of the effects of roads on amphibian and reptile populations, despite the fact that amphibians and reptiles have significantly more species at risk than mammals or birds. Additionally, available data of road-kills of amphibians and reptiles for Europe is scarce and often not comparable due to different study designs [16] and because of species-specific response patterns [17, 18].

In temperate regions like Central Europe many amphibian and some reptile species require complex landscapes including wetlands for reproduction and woody areas for foraging and hibernation [19]. Hence, the composition of landscape surrounding roads is an important factor influencing the number of road-kills for both amphibian and reptile populations [1, 20, 21]. Some reptile species (e.g. European green lizard, *Lacerta viridis*, Laurenti, 1768) are more selective to a habitat than other species (e.g. Grass snake, *Natrix natrix,* Linnaeus, 1758), which migrate long distances from summer to winter habitats. Most adult toads are susceptible to road-kills when migrating to breeding ponds [1, 10], while road-kills of juvenile toads are more dispersed in space and time when moving to hibernation sites in late summer and autumn. In the northern hemisphere most road-kill studies investigate the impact of wide roads with high traffic volumes that are usually fenced off and therefore are a stronger barrier to amphibians and reptiles [6, 22, 23]. However, evidence is increasing that tertiary road networks especially affect small animal species like herpetofauna [24–27]. Nevertheless, the influences of tertiary roads on amphibian and reptile populations on a landscape level are not often studied. When monitoring herpetofaunal road-kills challenges exist because small road-killed animals disappear quickly [28], the diverse network of tertiary roads is dense and some road-killed species are difficult to identify.

The standard approach to assess the effects of road-kill on animals is to collect data on a regular basis along certain routes [29], but this method is very cost-intensive and time consuming [30]. An alternative is to use a citizen science approach, i.e. involving citizens to report road-kill sightings [29, 31–34]. However, in most citizen science projects, “presence only” data are collected, which hamper proper statistical analyses of underlying factors for road-kills [35]. The aim of the current study was to test a systematic monitoring approach that would be appropriate to engage citizens in collecting presence and absence data. Results of this pilot study would be a proof-of-concept before engaging the general public, since many challenges (e.g. motivation of citizens) exist in establishing such a citizen science monitoring approach [36]. To test this approach, we used a citizen science software for monitoring road-killed amphibians and reptiles along a fixed bicycle tour on tertiary roads. Additionally, we examine the applicability of open access remote sensing data for a large-scale citizen science approach to describe spatial patterns of road-killed amphibians and reptiles on tertiary roads. The findings will be discussed with respect to their potential for designing a cost-effective large scale road-kill monitoring system based on a citizen science approach.

## Methods

We monitored road-kills for two activity periods of amphibians and reptiles from March 2014 to October 2015 on a 97.5-km stretch of road in a rural region in eastern Austria. We use the term *tertiary road network* to summarize (I) agricultural roads (used by farmers and cyclists, speed limit 30 km h^−1^), (II) cycling paths (used by cyclists only), (III) municipal roads (mainly used by residents, speed limit 50 km h^−1^) and (IV) interurban roads (mainly used by residents, speed limit 100 km h^−1^).

### Study area

**Fig. 1:**
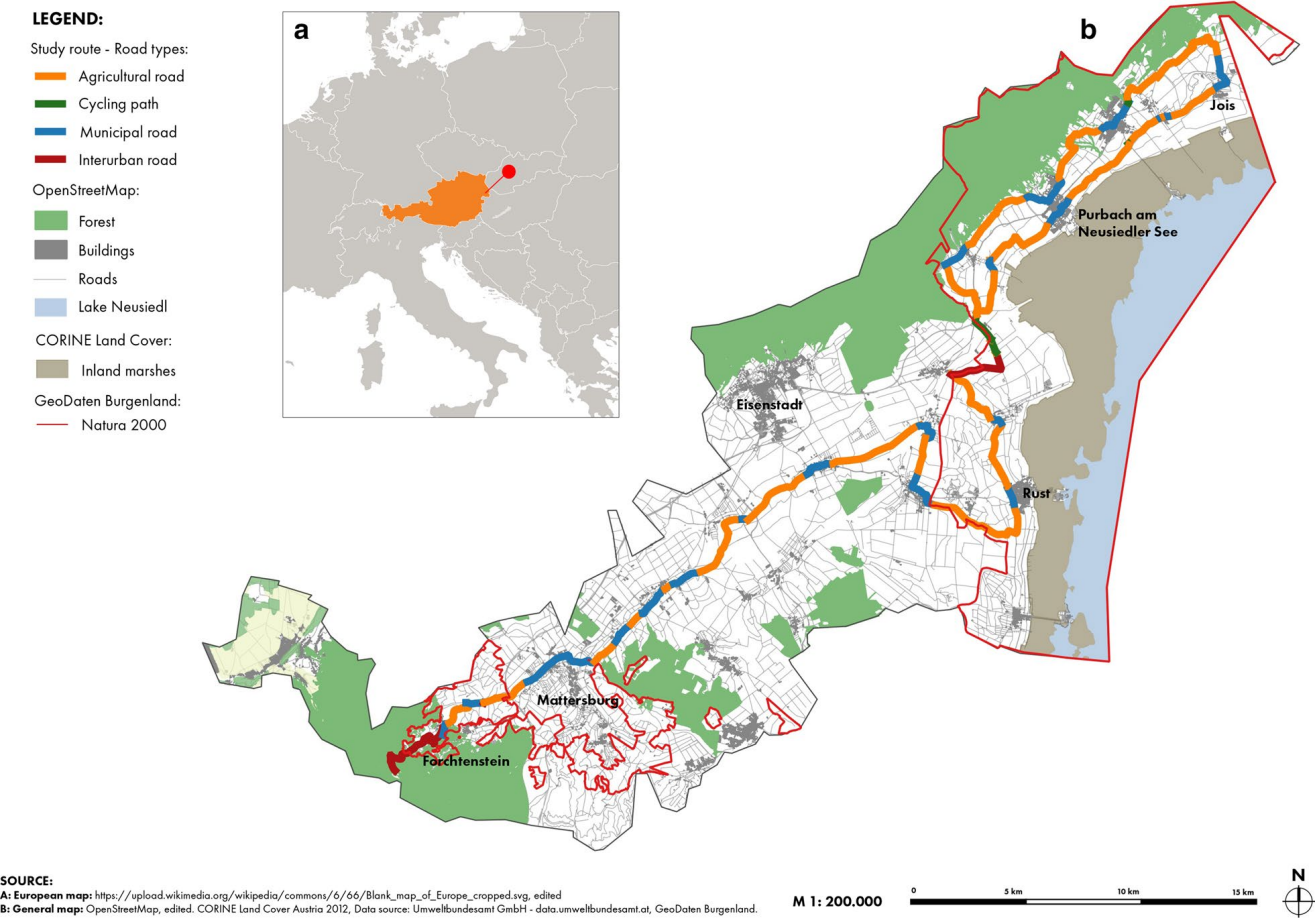
Location of the study route in Eastern Austria (**a**). Type of road sections of the study route (**b**)

The monitoring was conducted between the Leithagebirge and the Lake Neusiedl in Northern Burgenland, which is located in eastern Austria (Fig. 1). We chose the area for its high biodiversity in amphibian and reptile species and its relatively dense network of tertiary roads [37]. The study route consisted of 61 km of agricultural roads, 26 km of municipal roads, 8 km of interurban roads and 2.5 km of cycling paths, and is partly in the Natura 2000 sites Neusiedler See—Nordöstliches Leithagebirge (north-east of the study route) and Mattersburger Hügelland (south-west of the study route) [38]. The landscape of the study area is topographically heterogeneous with elevations ranging from 115 to 748 m. The climate of the region is considered Continental-Pannonian with 562 mm average annual precipitation and 10.7 °C annual mean temperature (Neusiedl am See; years 1981–2010) [39]. The Leithagebirge is forming the western boundary of the Natura 2000 site and at the same time framing the lake basin. The Leithagebirge exhibits a crystalline basement with accumulated reef lime stones of tertiary origin and is marked by mainly oak and mixed oak forests. In the outlying areas open farmland (vineyards interspersed with trees, bushes and grasslands residues) and dry grasslands dominate. The study area is a biodiversity hotspot in Austria as Pannonian, Alpine and Mediterranean floristic and faunal elements intermingle [38].

### Data collection

We monitored the selected route from March 2014 to October 2015 by using a smartphone app (Roadkill | SPOTTERON, NINC Media, Vienna, Austria) from the citizen science project Roadkill (http://www.roadkill.at/en). In the citizen science project volunteers collect presence only data of all vertebrate species killed on roads [35, 40]. The app was not adjusted to our approach but rather used as data collection tool in a more systematic way. In the current project, we used the Roadkill app to monitor road-killed amphibians and reptiles along a predefined route on average every 11 days. We monitored road-kills by cycling, since slower traveling speed results in higher detectability especially of smaller species [41]. We stopped at each road-killed animal and filled in the form provided by the SPOTTERON Roadkill application, which included coordinates of the spot, a picture of the animal, species name and number of individuals. All other locations where no road-kill was detected represent absence data. Our monitoring therefore results in a presence/absence dataset of locations along the route at different points in time.

### Remote sensing data

We used only open data to follow the idea of open science and to test the applicability of freely available remote sensing data in describing spatial patterns of road-killed amphibians and reptiles. We downloaded the most current CORINE (Coordination of Information on the Environment) land cover data from copernicus land cover monitoring services [42]. We divided the study route in 500 m sections and for each section assessed the surrounding landscape within a 500 m buffer on each side of the road by recording the area of each land cover class. The sum of the area of all land cover classes results in the study area. CORINE land cover (CLC) is a geographic land cover/land use database for a Pan-European region. CLC data provides information on the biophysical characteristics of the Earth’s surface based on images acquired by Earth Observation satellites with a Minimal Mapping Unit (MMU) of 25 ha [43]. The CLC 2012 uses a standardized European level-3 nomenclature consisting of 37 classes [44]. We used only the 14 classes which are present in the study area. The study area consisted of a high number of vineyards, arable land and urban areas (Table 1).

**Table 1:** CORINE land cover classes in the study area. Land cover classes in descending order of proportional area

Land cover class	CLC code	Area (ha)	Area (%)
Vineyards	221	3025.56	35.13
Non-irrigated arable land	211	2574.66	29.89
Discontinuous urban fabric	112	1809.06	21.00
Complex cultivation patterns	242	293.52	3.41
Land principally occupied by agriculture, with significant areas of natural vegetation	243	231.77	2.69
Broad-leaved forest	311	227.79	2.64
Pastures	231	127.98	1.49
Coniferous forest	312	119.28	1.38
Mixed forest	313	96.47	1.12
Continuous urban fabric	111	52.58	0.61
Industrial or commercial units	121	24.42	0.28
Sport and leisure facilities	142	14.12	0.16
Inland marshes	411	11.89	0.14
Transitional woodland shrub	324	3.91	0.05

### Statistical analyses

First, monthly variations of road-kills per year were analysed using Chi squared tests. Second, to find road-kill hotspots on the study route, we used the software KDE+ [45]. KDE+ identifies clusters of road-kills based on kernel density estimation and provides a measure of the significance of a hotspot based on Monte Carlo simulations [46]. Third, we divided the study route in 500 m sections to calculate conditional probabilities to assess the association of a certain land cover class with a road-kill event directly from the sample. Conditional probabilities [P(E|B)] of road-kill events (E) on each land cover class (B) were calculated to analyse which land cover classes are associated with an increased/decreased risk of road-kills, as compared to the overall probability P(E) of sections having a road-kill event. To obtain conditional probabilities, the areal fractions of land cover classes were determined for each section. The probability of a road-kill on a certain land cover class [P(E∩B)] is the total area of B of sections affected by road-kills [A(E∩B)] divided by the total area of all sections (A). [P(E∩B)] was finally divided by the overall availability (i.e., total area fraction) of this land cover class in the study area [P(B)] to obtain its conditional probability. Fourth, to analyse which land cover class is related to the number of road-kills we employed general linear models (GLM) with Poisson distributions. Possible collinearity was handled by means of stepwise model fitting and variance inflation factors (VIF). From all fitted models that do not contain predictors VIF >10, the one with the lowest value in the akaike information criterion (AIC) was chosen.

Note that conditional probabilities and general linear models are performed to 500 m road sections whereas the study route as a whole was used for the kernel density estimation. Chi squared tests and general linear models were performed using the “Rcmdr” package (R Commander Version 2.2–3) [47] in the open source program “R” (R version 3.2.4) [48].

### Cost efficiency estimation

A rough estimation of the cost efficiency of our citizen science approach was made to compare costs of our pilot study with a classical monitoring approach. Numbers used for the calculation are based on standard staff costs in Austria, one offer provided by an Austrian engineering office and one offer provided by the software company which developed apps for project *Roadkill*.

## Results

During our monitoring of 20 months, we found 252 road-killed animals (180 amphibians and 72 reptiles) comprising eight species (Table 2). Green toad (*Bufo viridis,* Laurenti, 1768), common toad (*Bufo bufo,* Linnaeus, 1758) and grass snake (*Natrix natrix*) were the dominating species of the investigated amphibian and reptile species, respectively. Most amphibians and reptiles were killed on agricultural roads.

**Table 2:** Numbers of road-killed amphibians and reptiles found from March 2014–October 2015 on monitored sections of municipal roads (26 km), cycle paths (2.5 km), agricultural roads (61 km) and interurban roads (8 km)

Species	Municipal road		Cycle path		Agricultural road		Interurban road	
	Rk	Rk km^−1^	Rk	Rk km^−1^	Rk	Rk km^−1^	Rk	Rk km^−1^
Green toad (*Bufo viridis*)	45	1.73	1	0.4	69	1.13	4	0.5
Common toad (*Bufo bufo*)	4	0.15	0	0	50	0.82	1	0.13
Agile frog (*Rana dalmatina*)	1	0.04	0	0	3	0.05	1	0.13
Tree frog (*Hyla arborea*)	0	0	0	0	1	0.02	0	0
Grass snake (*Natrix natrix*)	7	0.27	1	1	43	0.7	9	1.13
Lizards (*Lacertidae*)	1	0.04	0	0	4	0.07	1	0.13
Smooth snake (*Coronella austriaca*)	0	0	0	0	3	0.05	0	0
Blind worm (*Anguis fragilis*)	0	0	0	0	2	0.03	1	0.13
	58	2.23	2	0.8	175	2.87	17	2.13

Numbers of road-killed animals (Rk) and road-killed animals per kilometer (RK km^−1^)

**Fig. 2:**
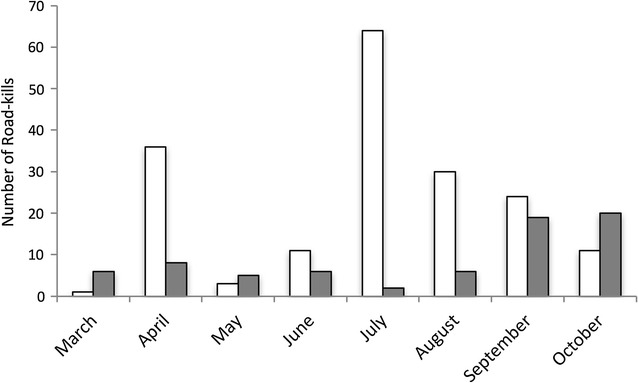
Total numbers of road-killed amphibians (*white*, n = 180) and reptiles (*grey*, n = 72) per month from March 2014–October 2015. No road-killed amphibians or reptiles were found between November 2014 and February 2015

Road-kill reports were not equally distributed across months (amphibians: X^2^ = 136.44, df = 7, p < 0.001; reptiles: X^2^ = 34.889, df = 7, p < 0.001). Figure 2 shows that most amphibians were reported in July (n = 64), followed by April (n = 36) and August (n = 30), whereas most reptiles were reported in October (n = 20) and September (n = 19).

### Spatial patterns

**Fig. 3:**
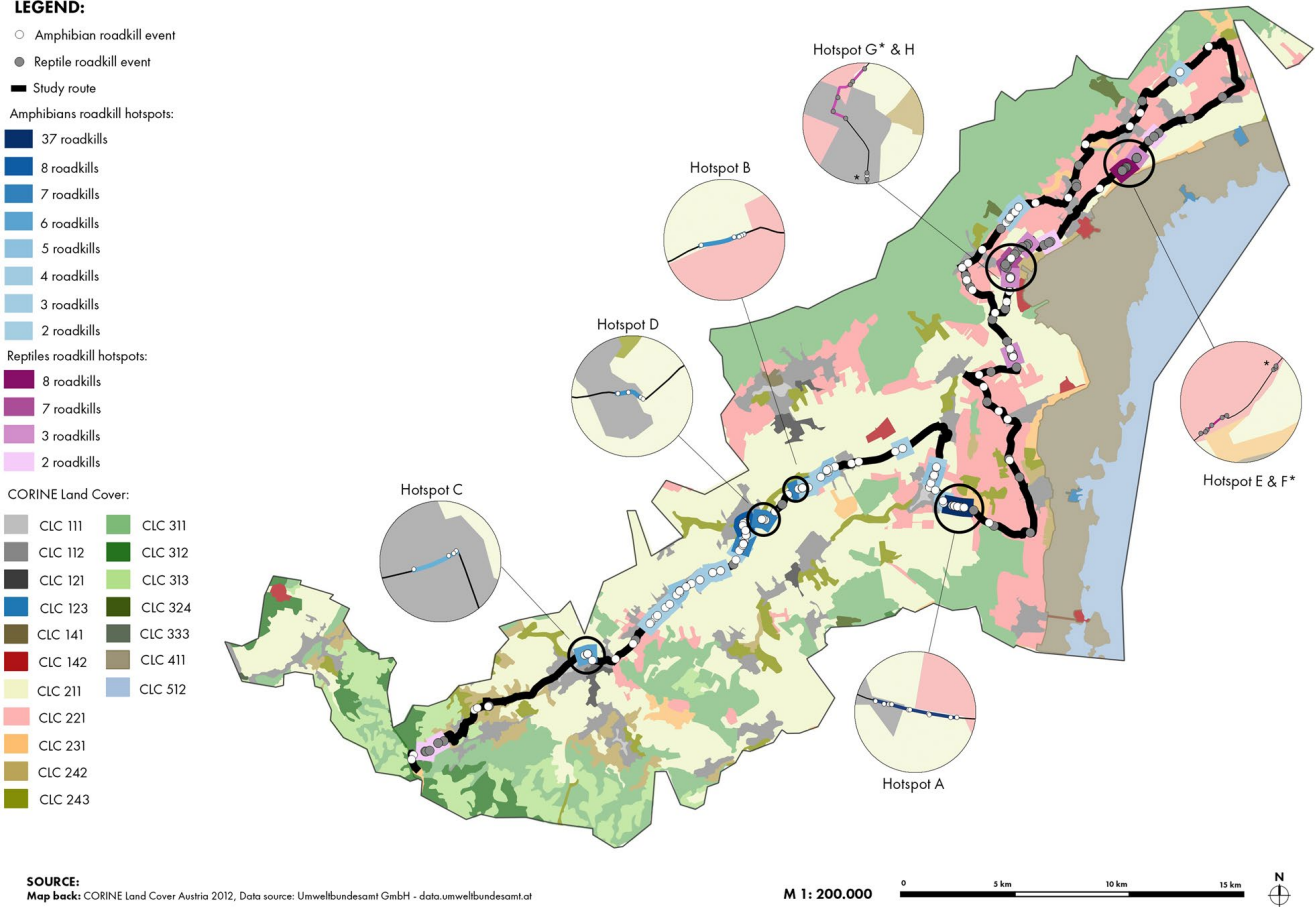
Amphibian (*blue*) and reptile (*purple*) road-kill hotspots calculated with KDE+. Highlighted are the four strongest hotspots of amphibians (*A*–*D*) and reptiles (*E*–*H*). *Asterisked letters* differ the two hotspots in one circle

Applying the KDE+ software to our road-kill monitoring data resulted in several hotspots including sections of 2–37 road-killed amphibians and of 2–8 road-killed reptiles, respectively (Fig. 3; Table 3). The vast majority of amphibian hotspots are next to arable land and suburban areas, whereas most reptile hotspots are located near the reed belt of the lake Neusiedl.

**Table 3:** KDE+ results of the strongest four amphibian and reptile hotspots

Animal group	Hotspot	Length (m)	Road-kills	Strength
Amphibians	A	523.49	37	0.96
	B	202.79	7	0.82
	C	152.77	6	0.79
	D	175.54	7	0.78
Reptiles	E	249.88	8	0.80
	F	36.29	3	0.66
	G	52.25	3	0.65
	H	532.09	7	0.58

Green toads and common toads represented 96% of all road-killed amphibians and grass snakes represented 83% of all road-killed reptile species, therefore we focused the following analyses on these three most often found species. Number of road-killed common toads and green toads per 500 m section varied from 0 to 35. The overall probability of a road-killed toad in the study area calculated per section was 0.36. This value was then used as a benchmark for conditional probabilities of road-kill events by land cover class (Table 4). All land cover classes with P(E|B) >0.36 are associated with increased risk of road-killed common toads and green toads, whereas land cover classes with P(E|B) <0.36 constrain the risk. Transitional woodland shrub (CLC 324), Sport and leisure facilities (CLC 142) and Land principally occupied by agriculture, with significant areas of natural vegetation (CLC 243) exhibit the highest conditional probabilities, whereas Industrial or commercial units (CLC 121) and Pastures (CLC 231) were associated with the lowest conditional probabilities. Using the best fitting general linear model focusing on the most often road-killed species common toad and green toad (AIC: 687.67; Table 5), a significant positive relation of Land principally occupied by agriculture, with significant areas of natural vegetation (CLC 243) and Transitional woodland shrub (CLC 324) and amphibian road-kills was seen, indicating that this land cover classes promoted amphibian road-kills. Complex cultivation patterns (CLC 242) and Vineyards (CLC 221) had a significantly negative relationship with the number of road-killed common toads and green toads.

**Table 4:** Conditional probabilities of road-killed common toads and green toads for all land cover classes [P(E|B)]

CLC code	Land cover class	H(E)	H(E1)	H(E)+ H(E1)	P(E∩B)	P(B)	P(E|B)
324	Transitional woodland shrub	0.088	0.000	0.088	0.000	0.000	0.998
142	Sport and leisure facilities	0.296	0.023	0.319	0.002	0.002	0.924
243	Land principally occupied by agriculture, with significant areas of natural vegetation	3.181	2.081	5.262	0.016	0.027	0.606
311	Broad-leaved forest	2.147	2.998	5.145	0.011	0.026	0.416
211	Non-irrigated arable land	23.681	34.538	58.220	0.121	0.299	0.406
112	Discontinuous urban fabric	16.534	24.576	41.110	0.085	0.210	0.404
411	Inland marshes	0.088	0.180	0.269	0.000	0.001	0.327
312	Coniferous forest	0.862	1.831	2.694	0.004	0.014	0.319
221	Vineyards	20.752	47.589	68.340	0.106	0.351	0.303
111	Continuous urban fabric	0.334	0.854	1.188	0.002	0.006	0.281
242	Complex cultivation patterns	1.742	4.950	6.692	0.009	0.034	0.262
313	Mixed forest	0.138	2.094	2.232	0.001	0.011	0.063
231	Pastures	0.157	2.734	2.891	0.001	0.015	0.054
121	Industrial or commercial units	0.000	0.552	0.552	0.000	0.003	0.000

Proportion of each land cover class per section containing road-killed toads [H(E)] or not [H(E1)]. The probability of a road-killed toad on a certain land cover class [P(E∩B)] divided by the overall availability of this land cover class in the study area [P(B)] results in the conditional probability P(E|B). Land cover classes in descending order of P(E|B); the higher the P(E|B), the higher the probability of a road-kill on the specific land cover class

**Table 5:** GLM containing land cover classes as explanatory variables that influence road-kill numbers of green toad and common toad

CLC code	Land cover class	VIF	Estimate	Std. error	z value	P(>|z|)
Intercept			3.53E-01	1.51E-01	2.342	0.019
243	Land principally occupied by agriculture, with significant areas of natural vegetation	1.377	1.05E-05	2.26E-06	4.667	3.06E-06
242	Complex cultivation patterns	1.259	−1.56E-05	3.57E-06	−4.374	1.22E-05
221	Vineyards	1.276	−2.43E-06	6.12E-07	−3.964	7.38E-05
324	Transitional woodland shrub	1.086	5.30E-05	2.32E-05	2.284	0.022
231	Pastures	1.049	−1.47E-05	8.20E-06	−1.795	0.073
111	Continuous urban fabric	1.019	−1.52E-05	8.58E-06	−1.778	0.076
313	Mixed forest	1.14	−2.17E-05	1.46E-05	−1.486	0.137
311	Broad-leaved forest	1.176	−2.76E-06	3.35E-06	−0.825	0.41
142	Sport and leisure facilities	1.024	−7.70E-06	1.25E-05	−0.617	0.537
312	Coniferous forest	1.186	−1.22E-06	2.99E-06	−0.407	0.684
411	Inland marshes	1.041	−1.20E-05	3.21E-05	−0.372	0.71
112	Discontinuous urban fabric	1.283	−1.16E-07	6.47E-07	−0.179	0.858
121	Industrial or commercial units	1	−2.15E-04	9.17E-03	−0.023	0.981

Land cover classes in descending order of P(>|z|)

Number of road-killed grass snakes per 500 m section varied from 0 to 6, with an overall probability of a road-killed grass snake per section of 0.17. Again, conditional probabilities P(E|B) were calculated for all 14 land cover classes (Table 6). Inland marshes (CLC 411) and Sport and leisure facilities (CLC 142) appeared to promote reptile road-kills, whereas Transitional woodland shrub (CLC 324), Industrial or commercial units (CLC 121) and Continuous urban fabric (CLC 111) appeared to constrain them. Using the best fitting general linear model again excluding all but the most often road-killed reptile species grass snake (AIC: 294.79; Table 7), resulted in significantly positive relations of the land cover classes Inland marshes (CLC 411), Sport and leisure facilities (CLC 142) and Complex cultivation patterns (CLC 242) with reptile road-kills. Discontinuous urban fabric (CLC 112) and Land principally occupied by agriculture, with significant areas of natural vegetation (CLC 243) were significantly negative related to the number of road-killed grass snakes.

**Table 6:** Conditional probabilities of road-killed grass snakes for all land cover classes [P(E|B)]

CLC code	Land cover class	H(E)	H(E1)	H(E)+ H(E1)	P(E∩B)	P(B)	P(E|B)
411	Inland marshes	0.143	0.126	0.269	0.001	0.001	0.531
142	Sport and leisure facilities	0.147	0.172	0.319	0.001	0.002	0.459
242	Complex cultivation patterns	2.191	4.501	6.692	0.011	0.034	0.330
311	Broad-leaved forest	1.296	3.849	5.145	0.007	0.026	0.251
211	Non-irrigated arable land	11.749	46.471	58.220	0.060	0.299	0.202
221	Vineyards	12.197	56.143	68.340	0.063	0.351	0.178
313	Mixed forest	0.380	1.851	2.232	0.002	0.011	0.174
243	Land principally occupied by agriculture, with significant areas of natural vegetation	0.900	4.362	5.262	0.005	0.027	0.172
231	Pastures	0.393	2.497	2.891	0.002	0.015	0.136
312	Coniferous forest	0.286	2.408	2.694	0.001	0.014	0.106
112	Discontinuous urban fabric	4.318	36.792	41.110	0.022	0.210	0.105
111	Continuous urban fabric	0.000	1.188	1.188	0.000	0.006	0.000
121	Industrial or commercial units	0.000	0.552	0.552	0.000	0.003	0.000
324	Transitional woodland shrub	0.000	0.088	0.088	0.000	0.000	0.000

Proportion of each land cover class per section containing road-killed grass snakes [H(E)] or not [H(E1)]. The probability of a road-killed grass snake on a certain land cover class [P(E∩B)] divided by the overall availability of this land cover class in the study area [P(B)] results in the conditional probability P(E|B). Land cover classes in descending order of P(E|B); the higher the P(E|B), the higher the probability of a road-kill on the specific land cover class

**Table 7:** GLM containing land cover classes as explanatory variables that influence road-kill numbers of grass snakes

CLC code	Land cover class	VIF	Estimate	Std. error	z value	P(>|z|)
Intercept			−8.82E-01	2.82E-01	−3.129	0.002
411	Inland marshes	1.243	5.95E-05	1.22E-05	4.894	9.90E-07
142	Sport and leisure facilities	1.142	2.42E-05	1.03E-05	2.35	0.019
242	Complex cultivation patterns	1.579	5.81E-06	2.57E-06	2.262	0.024
243	Land principally occupied by agriculture, with significant areas of natural vegetation	1.483	−1.31E-05	6.32E-06	−2.08	0.038
112	Discontinuous urban fabric	1.277	−3.08E-06	1.49E-06	−2.067	0.039
221	Vineyards	1.447	−1.21E-06	1.08E-06	−1.118	0.264
312	Coniferous forest	1.099	−5.08E-06	5.79E-06	−0.878	0.38
231	Pastures	1.189	1.28E-06	3.68E-06	0.347	0.729
311	Broad-leaved forest	1.154	1.83E-07	4.09E-06	0.045	0.964
313	Mixed forest	1.113	2.40E-07	5.78E-06	0.042	0.967
111	Continuous urban fabric	1	−2.81E-03	2.24E-01	−0.013	0.989
324	Transitional woodland shrub	1	−1.66E-03	8.79E-01	−0.002	0.999
121	Industrial or commercial units	1	−2.71E-04	1.56E-01	−0.002	0.999

Land cover classes in descending order of P(>|z|)

### Cost efficiency estimation

We compared two cases for cost-efficiency estimation (Table 8), monitoring by researchers only and a citizen science approach. Based on our calculations, using an approach involving researchers only would result in 22,000 €. This includes monitoring road-kills by a graduate student on a study route of 100 km over two vegetation periods and recording the surrounding landscape structure and habitat descriptions by an engineering office. Alternatively, the use of citizen science in combination with open access land cover data as we supposed in our pilot study would result in 86,000 €. This includes adjusting the citizen science smartphone application of the project *Roadkill* to allow for monitoring roads, maintaining the application and professional support of the participants for a 2-year period.

**Table 8:** Cost-efficiency estimation for the cases researcher and citizen science

Cases	Costs (€)
Researcher	
Road-kill monitoring	10,000
Assessing surrounding land cover	12,000
Total	22,000
Citizen science	
App adjustment	20,000
App and website maintenance	16,000
Professional support	50,000
Total	86,000

Costs are calculated in Euro for monitoring 100 km of roads over 2 years

## Discussion

To our knowledge, this is among the first studies testing the suitability of a citizen science approach in examining the impact of tertiary roads and the surrounding landscape on amphibian and reptile species by using freely-available remote sensing data.

Road-kills of both amphibians and reptiles were recorded during the whole vegetation period mainly on agricultural roads. The role of tertiary roads for road-kill of endangered species is generally not widely appreciated, however is perhaps more significant in regions where higher ranking roads are already equipped with efficient road-kill mitigation measures. Green toads and common toads represented 96% of all road-killed amphibians with peaks in April, August and September. These two species are also among the most abundant species in the study region [37]. From our results it seems that identifying road-kill hotspots of both species in spring would be most straightforward because of mass migration to the spawning sites during this time [49]. However, when the goal is to assess the effect of road-kill on population dynamics, surveys would need to also include surveys in late summer and autumn when individuals of both species forage in their terrestrial habitat up to 10 km away from the breeding ponds [50]. Grass snakes represented 83% of all road-killed reptile species with road-kill peaks in September and October. Grass snake is the most abundant snake species in the study region [37]. Starting in November, Grass snakes overwinter underground in areas which are not subject to freezing (e.g. compost heaps, burrows of mice) and get active again in March or April [37]. One reason for the peaks of road-killed reptiles in September and October could be that young grass snakes hatch from their nesting sites in August and sprawl into the surrounding landscape crossing our study route. Indeed, when we double-checked the photos of the road-killed reptiles it turned out that about 80% of road-killed grass snakes were juvenile. Additionally, in September and October roads are frequently used by snakes and lizards for basking during the day, but roads during these months are also more frequently used by farmers during wine harvest. Numbers of both amphibian and reptile road-kills per month suggest, that road-kill monitoring should comprise whole activity periods to get a complete overview. Here citizen science would be a suitable approach, since it would be very costly to monitor amphibians and reptiles on tertiary roads covering a wide geographic range in short time periods with a classic approach involving researchers only.

Additionally, KDE+ analyses showed significant road-kill hotspots indicating that road-kills are not randomly distributed in the landscape. This is an important information for nature conservation authorities aiming to mitigate threats for endangered amphibian and reptile species.

Conditional probabilities and general linear models applied in our study showed a positive relationship of the land cover classes Transitional woodland shrub (CLC 324) and Land principally occupied by agriculture, with significant areas of natural vegetation (CLC 243) and common toad and green toad road-kills. It was encouraging to see, that land cover classes based on a rather coarse 500-m grid matched well with the most preferred habitats of the most abundant amphibian species in the study. Green toads and common toads use water bodies only for spawning. Green toads inhabit various kinds of terrestrial sites including gravel pits, field edges, ruderal plots, dry grassland, open forests or suburban areas whereas common toads live mainly in areas with dense vegetation such as forests and scrubland areas, parks or gardens [37]. Grass snake road-kills were positive related to land cover classes Inland marshes (CLC 411), Sport and leisure facilities (CLC 142) and Complex cultivation patterns (CLC 242). Grass snakes, as the most frequently found reptile species in our study inhabit a broad range of open or semi-open habitats, including reed belts, riparian zones, forests, gardens or parks. Grass snakes can be found in the reed belt of the lake Neusiedl in high numbers matched by the CORINE land cover class Inland marshes [37]. We monitored the study route on average every 11 days and might have underestimated the number of road-kills as the persistence time of carcasses could be lower [28, 51]. Notwithstanding this limitation, our current results are in line with previous studies. This is especially important, more frequent monitorings would be difficult over a long time span with a citizen science approach. Generally, the suitability of the CORINE land cover dataset for modelling amphibian and reptile road-kills encourages us to apply our monitoring approach to a broader geographical scale.

Based on our rough cost efficiency estimation, monitoring the influence of land cover on road-kills with a citizen science approach is suitable when monitoring more than 400 km road sections. Below 400 km a conventional monitoring approach with professional researchers only seems to be more efficient. However, this calculation is just a rough estimation and should be treated with care; it is based on Austrian standard staff costs and is calculated for investigating the factor land-cover only. If other factors besides land cover are planned to be investigated, the cost efficiency could be tremendously different.

## Conclusions

Overall, our findings confirmed previous results showing that amphibian and reptile species are especially susceptible to road-kill in the vicinity of their preferred habitats [1, 52]. Nevertheless, this is interesting, as we achieved these results using freely available remote sensing data and a survey technique that could easily be adopted on a larger scale using a citizen science approach. We are confident, that the results of this pilot study can be used as basis for other citizen science projects in this field trying to enlarge their study area. A first step to the extension of our monitoring system would be to get an overview of road-killed amphibians and reptiles on a landscape scale by monitoring tertiary road networks potentially using a citizen science approach to cover this wide geographic range [35, 36, 53]. Furthermore, these data could then be used to reduce the impact of road traffic on amphibians and reptiles by installing temporal or permanent mitigation measures.

## Abbreviations

AIC: (akaike information criterion)
CLC: (CORINE land cover)
CORINE: (coordination of information on the environment)
GLM: (general linear model)
KDE+: (kernel density estimation plus)
VIF: (variance inflation factors)
WMTS: (web map tile service)
